# CDK4/6 Inhibitor Treatments in Patients with Hormone Receptor Positive, Her2 Negative Advanced Breast Cancer: Potential Molecular Mechanisms, Clinical Implications and Future Perspectives

**DOI:** 10.3390/cancers13020332

**Published:** 2021-01-18

**Authors:** Michela Roberto, Antonio Astone, Andrea Botticelli, Luisa Carbognin, Alessandra Cassano, Giuliana D’Auria, Agnese Fabbri, Alessandra Fabi, Teresa Gamucci, Eriseld Krasniqi, Mauro Minelli, Armando Orlandi, Francesco Pantano, Ida Paris, Laura Pizzuti, Ilaria Portarena, Nello Salesi, Simone Scagnoli, Paola Scavina, Giuseppe Tonini, Patrizia Vici, Paolo Marchetti

**Affiliations:** 1 Oncology Unit, Department of Clinical and Molecular Medicine, Sant’Andrea Hospital, Sapienza University of Rome, Via di Grottarossa 1035-1039, 00189 Rome, Italy; paolo.marchetti@uniroma1.it; 2 Division of Medical Oncology, Fatebenefratelli San Pietro Hospital, 00189 Rome, Italy; astone.antonio@fbfrm.it; 3 Medical Oncology Unit B, Policlinico Umberto I, 00161 Rome, Italy; Andrea.botticelli@uniroma1.it; 4 Department of Woman and Child Health and Public Health, Fondazione Policlinico Universitario A. Gemelli IRCCS, 00168 Rome, Italy; luisa.carbognin@guest.policlinicogemelli.it (L.C.); ida.paris@policlinicogemelli.it (I.P.); 5 Department of Medical Oncology, Catholic University of Sacred Heart, 00168 Rome, Italy; Alessandra.cassano@policlinicogemelli.it (A.C.); armando.orlandi@policlinicogemelli.it (A.O.); 6 Medical Oncology, Sandro Pertini Hospital, 00157 Rome, Italy; Giuliana.dauria@aslroma2.it (G.D.); teresa.gamucci@aslroma2.it (T.G.); 7 Medical Oncology Unit, Belcolle Hospital, 01100 Viterbo, Italy; mariaagnese.fabbri@asl.vt.it; 8 Phase 1 Unit and Pre+cision Medicine, IRCCS Regina Elena National Cancer Institute, 00144 Rome, Italy; alessandra.fabi@policlinicogemelli.it; 9 Division of Medical Oncology 2, IRCCS Regina Elena National Cancer Institute, 00144 Rome, Italy; eriseld.krasniqi@ifo.gov.it (E.K.); laura.pizzuti@ifo.gov.it (L.P.); patrizia.vici@ifo.gov.it (P.V.); 10 San Giovanni Addolorata Hospital, 00184 Rome, Italy; mminelli@hsangiovanni.roma.it (M.M.); paola.scavina@hsangiovanni.roma.it (P.S.); 11 Department of Oncology, University Campus Biomedico of Rome, 00155 Rome, Italy; F.pantano@unicampus.it (F.P.); g.tonini@unicampus.it (G.T.); 12 Medical Oncology Unit, Department of Systems Medicine, Tor Vergata Clinical Center University Hospital, 00133 Rome, Italy; ilaria.portarena@ptvonline.it; 13 Medical Oncology, S.M. Goretti Hospital, 04100 Latina, Italy; n.salesi@ausl.latina.it; 14 Department of Medical and Surgical Sciences and Translational Medicine, Sapienza University of Rome, 00185 Rome, Italy; simone.scagnoli@uniroma1.it

**Keywords:** CDK4/6 inhibitors, breast cancer, endocrine therapy (ET), advanced breast cancer (ABC), endocrine resistance

## Abstract

**Simple Summary:**

The recent addition of cyclin-dependent kinase 4 (CDK4) and CDK6 inhibitors (palbociclib, ribociclib, abemaciclib) to endocrine therapy have remarkably improved the outcome of patients with HR+ advanced breast cancer. However, some points of reflections are still undiscussed. To answer these questions, we revised the mechanism of action of CDK4-6 inhibitors, clinical data available from pivotal studies, and summarized potential future strategies to overcome resistance to CDK4-6 inhibitors, thus improving patient’s survival.

**Abstract:**

Hormone receptor (HR)-positive, human epidermal growth factor receptor 2 (HER2)-negative breast cancer is the most common breast cancer subtype, and endocrine therapy (ET) remains its therapeutic backbone. Although anti-estrogen therapies are usually effective initially, approximately 50% of HR+ patients develop resistance to ET within their lifetime, ultimately leading to disease recurrence and limited clinical benefit. The recent addition of cyclin-dependent kinase 4 (CDK4) and CDK6 inhibitors (palbociclib, ribociclib, abemaciclib) to ET have remarkably improved the outcome of patients with HR+ advanced breast cancer (ABC) compared with anti-estrogens alone, by targeting the cell-cycle machinery and overcoming some aspects of endocrine resistance. However, which patients are the better candidates for these drugs, which are the main characteristics for a better selection of patients or if there are predictive biomarkers of response, is still unknown. In this review we reported the mechanism of action of CDK4/6 inhibitors as well as their potential mechanism of resistance, their implications in clinical practice and the forthcoming strategies to enhance their efficacy in improving survival and quality of life of patients affected with HR+, HER2−, ABC.

## 1. Introduction

Hormone receptor (HR)-positive, human epidermal growth factor receptor 2 (HER2)-negative breast cancer is the most common breast cancer subtype, and endocrine therapy (ET) remains its therapeutic backbone. Although anti-estrogen therapies are usually effective initially, approximately 50% of HR+ patients develop resistance to ET within their lifetime, ultimately leading to disease recurrence and limited clinical benefit [1]. The recent addition of cyclin-dependent kinase 4 (CDK4) and CDK6 inhibitors (palbociclib, ribociclib, abemaciclib) to ET have remarkably improved the outcome of patients with HR+ advanced breast cancer (ABC) compared with anti-estrogens alone, by targeting the cell-cycle machinery and overcoming some aspects of endocrine resistance.

### 1.1. Mechanism of Endocrine Therapy Resistance

**Figure 1:**
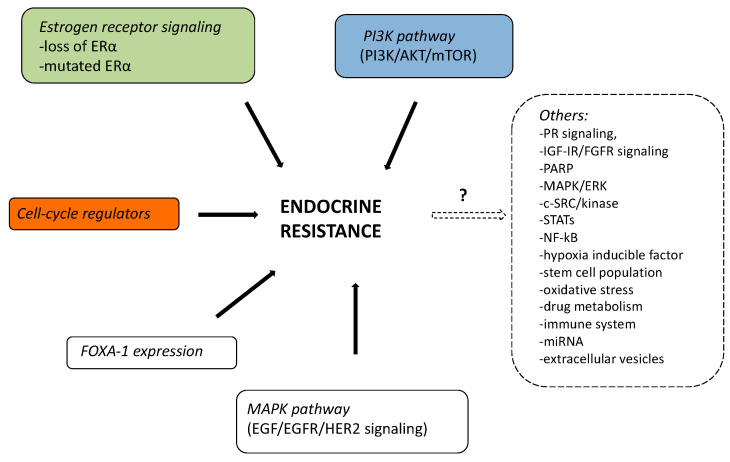
Possible mechanisms of endocrine resistance in summary.

Until now, three distinct pathways of regulation of estrogen receptor (ER) gene (*ESR1*) expression were thought to be mainly involved in breast cancer resistance to ET [2]: (i) Classic signaling: ligand-binding domain mutations in the ER that activates *ESR1* expression (approximately 18% of endocrine-resistant HR+ breast cancers); (ii) Ligand independent signaling: ER can also be activated as a consequence of signaling events downstream of receptor tyrosine kinases (RTKs); (iii) Non-genomic mechanisms: signaling can be mediated by ER that is localized at the cell membrane or in the cytoplasm of breast cancer cells. A figure that summarizes all the described endocrine-resistance mechanisms was reported. (Figure 1)

#### 1.1.1. Mutations of ER-α

*ER* mutations are rare in primary tumors but appear to be reasonably frequent in the progression to endocrine resistance [3]. The spot mutations drive estrogen-independent transcriptional activity and cancer cell proliferation, leading to endocrine resistance [4].

#### 1.1.2. Loss of ER-α

Lack of *ER* is one of the principal causes of de novo resistance to ET. The loss of *ER-α* expression can be achieved by epigenetic mechanisms such as methylation of CpG islands or histone deacetylase activity in the *ESR1* promoter; DNA methyltransferase (DMNT) and histone deacetylase (HDAC) influence chromatin condensation regulating the *ER* gene expression [5].

In vitro experiments showed that DNMT1 inhibitors (Aza) and HDAC inhibitors (TSA) reduce chromatin condensation leading to ER expression in ER-negative breast cancer cells [6]. Moreover, AZA + TSA treatment inhibits tumor growth in mice inoculated with ER-negative breast cancer cells after ovarian ablation and restores sensitivity to tamoxifen [7].

#### 1.1.3. MAPK Pathway (EGF/EGFR/HER2 Signaling)

Mutation in the MAPK pathway has been reported in approximately 13% of breast cancers [1]. In addition to the expected *ESR1* hotspot mutations, *ERBB2* and *NF1* were the genes, mostly mutually exclusive, with the greatest difference in mutational frequency between pre- and post-hormonal therapy for HR + HER2− breast cancers [1]. Tamoxifen-resistant breast cancer cells (LTam) showed an hyperactivation of the *HER/EGFR/Akt/ERK* pathway. An in vitro study demonstrated that, by using lapatinib, a dual inhibitor of *EGFR* and *HER2*, tamoxifen sensitivity of LTam cells was restored [8].

#### 1.1.4. PI3K Pathway (PI3K/AKT/mTOR)

Resistance to letrozole in breast cancer cells is associated with hyperactivation of *p70S6K* and *AKT*, which are involved in the PI3K pathway. PI3K inhibitor (BEZ235, AEW541), mTOR inhibitor (RAD001), and EGFR/HER2 inhibitor (lapatinib) suppress proliferation of letrozole-resistant breast cancer cells [9]. *PI3K* inhibition enhances *ER* function and the response to endocrine therapies. Indeed, the PI3K inhibitor alpelisib (BYL719) in combination with the ER inhibitor fulvestrant has profound antitumor activity both in vivo and in vitro [10].

#### 1.1.5. FOXA-1 Expression

Foxa-1 is an essential protein for the transcriptional activity of both ER and androgen receptor (AR). The induction of *FOXA-1* expression with doxycycline in breast cancer cells was directly related to a high level of expression of proliferation genes and inversely to estrogen sensitivity genes. Moreover, increased expression of *FOXA1* contributes to tumor aggressiveness and endocrine resistance [11].

Other genomic and nongenomic mechanisms of resistance to ET are under investigation (eg, progesterone receptor signaling, IGF-IR, FGFR signaling, PARP, MAPK/ERK, c-SRC/KINASE, STATs, NF-kB, hypoxia inducible factor, stem cell population, oxidative stress, drug metabolism, immune system, miRNA, and extracellular vesicles) although the precise mechanisms remain largely unexplained. Complicating matters, some patients with ABC have distinct and coexisting mechanisms of resistance to ET in distinct tumor subclones that cannot be captured by a single biopsy of a metastatic site. Rizavi et al. [1] suggest that there was an emerging taxonomy of endocrine-resistant breast cancer, but some of these alterations were a consequence of selective therapeutic pressure and mechanisms of systemic therapy resistance. Therefore, to better define the complexity of endocrine resistance in HR+, HER- ABC, further genomic study of a large cohort of clinically phenotyped patients is needed.

#### 1.1.6. Cell-Cycle Regulators and Endocrine Resistance

Activation by D-type cyclin proteins leading to phosphorylation of retinoblastoma-associated protein and E2F protein-mediated transcription of cell-cycle genes, such as cyclins A and E, are critical for cell-cycle progression. Therefore, the action of cyclin-dependent kinases 4/6 (CDK4/6) by regulating the transition from G1-to-S cell-cycle phase is crucial for normal and cancer cell proliferation [12].

Indeed, CDK4/6 inhibitors have shown significant preclinical activity in ER-positive breast cancer, especially when combined with anti-estrogen therapy. In an in vitro experiment, CDK4 inhibitor (PD-0332991) reduced cell tumor growth of fulvestrant-insensitive ER+ cell lines and tumor growth of mice bearing an ER+ breast cancer cell line [13].

On transcriptome analysis, 58 tumor samples from letrozole-resistant patients were enriched for cell-cycle related genes. Treatment with palbociclib compared with fulvestrant significantly downregulated the expression of cell-cycle genes associated with letrozole resistance [14].

Abemaciclib is also a highly selective, reversible CDK 4/6 inhibitor with the highest half maximal inhibitory concentrations of 2 nM and 10 nM for CDK4 and CDK6, respectively [15].

In addition, ribociclib showed remarkable preclinical efficacy in ER+ BC mouse models by reducing tumor growth both as a single agent and in combination with letrozole or fulvestrant and with a PI3K inhibitor.

Although the three approved CDK4/6 inhibitors—palbociclib, ribociclib, and abemaciclib—seem to have essentially overlapping patterns of activity, as multikinase inhibitors, they could have many other mechanisms of action on several cellular populations other than tumor cells, particularly in the bone microenvironment. The extent to which these off-target events occur may also explain the difference in survival reported with the three different CDK4/6 inhibitors, but their significance in the overall treatment of disease is still not clear [16].

### 1.2. Clinical Implications

At the 4th ESO-ESMO international consensus, primary endocrine resistance was defined as “relapse while on the first 2 years of adjuvant ET, or PD within first 6 months of first-line ET for ABC, while on ET” and secondary endocrine resistance as “relapse while on adjuvant ET but after the first 2 years, or relapse within 12 months of completing adjuvant ET, or PD ≥ 6 months after initiating ET for ABC, while on ET”. However, these definitions are subsequent to CDK4/6 inhibitors trials and limited to 67% consensus.

According to endocrine sensitivity/resistance, four main scenarios are represented among the phase 3 CDK4/6-based trials: (1) de novo metastatic disease; (2) late relapse; (3) early relapse; and (4) second line (Table 1).

#### 1.2.1. First Line

According to the literature data, international clinical guidelines recommended ET as the preferred option for HR+, HER2− ABC in as first-line therapy, even in the presence of visceral disease.

Chemotherapy is reserved for visceral crisis, defined as severe organ dysfunction and rapid progression of disease, or progression on multiple lines of ET. A recent metanalysis showed that no chemotherapy regimen with or without targeted therapy is significantly better than CDK4/6 inhibitors plus hormone therapies in terms of progression-free survival [26]. Thus, considering the significant improvement in the outcome of patients with HR+, HER2− ABC with adjunct of CDK4/6 inhibitor to standard hormonal therapies compared with ET alone, the combinatorial strategy of CDK4/6 inhibitors and ET should be considered as the new standard of care in first- or second-line therapy (Table 1). A network meta-analysis, including patients treated with CDK4/6 inhibitors combined with aromatase inhibitors (AIs) or fulvestrant in comparison with AI or fulvestrant monotherapy, confirmed CDK4/6 inhibitors had similar efficacy when associated with an AI in the first-line treatment of HR+ ABC, and were superior to either fulvestrant or AI monotherapy, regardless of any other patient or tumor characteristics [27].

In de novo patients, all the CDK4/6 inhibitors performed better than ET alone in terms of progression-free survival (PFS) (MONALEESA-2, hazard ratio, 0.45; MONALEESA-3, hazard ratio, 0.57; MONALEESA-7, hazard ratio, 0.43; MONARCH-3, hazard ratio, 0.54; PALOMA-2, hazard ratio, 0.67) [17,18,19,20,22]. The MONALEESA-7 trial was the only phase 3 trial to study CDK4/6 inhibitors as first-line therapy in a premenopausal population; the percentages of premenopausal patients studied with palbociclib and abemaciclib derived from the second-line trials, PALOMA-3 and MONARCH-2, were 20.7% and 16.1%, respectively, where CDK4/6 inhibitors were combined with fulvestrant. The updated analysis of MONALEESA-7 showed that the addition of ribociclib to ET significantly prolonged overall survival (OS) compared with ET alone with an estimated OS at 42 months of 70.2% in the ribociclib group and 46.0% in the placebo group (hazard ratio, 0.71; *p*  =  0.00973) [28]. On the basis of its innovative results, ribociclib plus ET (AI/TAM) with ovarian function suppression (OFS) was recently approved by the Italian Medicines Agency (AIFA), and thus it could be considered as the preferred first-line treatment option in premenopausal patients with HR+, HER2− ABC. The overall response rate (ORR) was similar in MONALEESA-2 (ORR = 52.7%), MONARCH-3 (ORR= 59.2%), and PALOMA 2 (ORR = 55.3%). However, with a median PFS of 33.6 months rather than 22–28 months with palbociclib or with abemaciclib plus AI, and a relative risk reduction in death of 28% in the first-line setting of MONALEESA-3, ribociclib plus fulvestrant seems to be the preferred first-line treatment option in postmenopausal patients [19]. Moreover, whereas the data on OS for palbociclib and abemaciclib are still immature or are from real-world data of retrospective studies [29,30], the data on OS with ribociclib come directly from phase 3 trials [18,19].

The three CDK4/6 inhibitors reported a good toxicity profile; there was higher incidence of grade 3–4 neutropenia with both ribociclib and palbociclib, and diarrhea and abdominal pain with abemaciclib (Table 1). QTcF prolongation with ribociclib occurred in no more than 23 patients (7% of cases) in the MONALEESA-7 trial. However, the percentage of cases of QT prolongation was even lower in clinical practice. Dose reductions due to adverse events was reported in 54.5%, 33.1%, and 31% of cases in MONALEESA-2, MONALEESA-3, and MONALEESA-7, without any significant impact on PFS [17,18,19]. Thus, if toxicity occurs, the dosage of ribociclib can be reduces without affecting its efficacy. Both MONARCH [20,21] and PALOMA-3 [24] studies showed no difference in PFS for patients who had the dose reduced due to any adverse events compared with those who did not.

**Figure 2:**
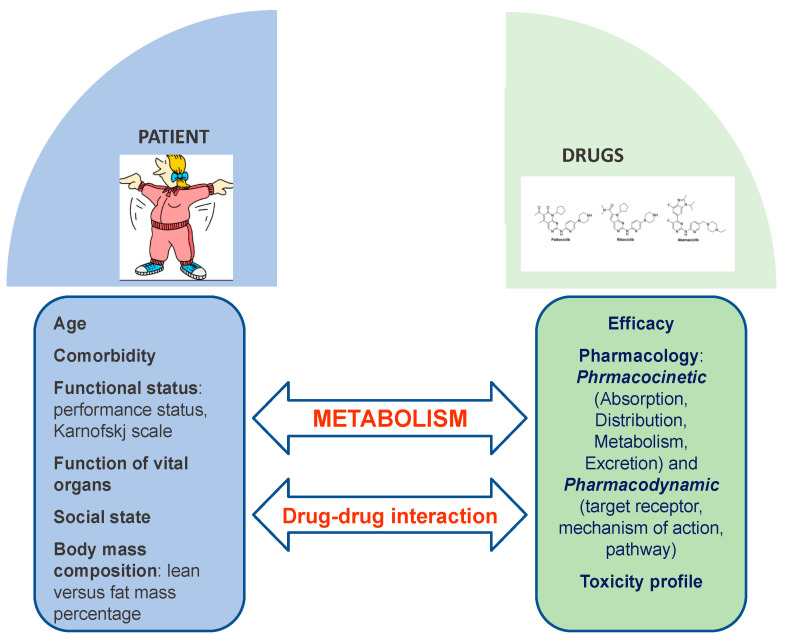
The complexity of patient metabolism and potential drug-drug-interactions.

However, the clinical scenario could be much more complex according to potential drug-drug interactions (DDIs) in patients with breast cancer treated with CDK4/6 inhibitors; DDIs may occur in patients who take polypharmacy [31]. Therefore, better knowledge of how patient metabolism and DDIs could affect both the efficacy and safety of CDK4/6 inhibitors should always be considered to maximize the personalization of cancer care in patients with ABC (Figure 2). Indeed, any physician should know that concomitant medications (e.g., proton pump inhibitors and corticosteroids), pharmacogenetic profile, and pathophysiological conditions could influence absorption, distribution, metabolism, and elimination pharmacokinetics. A personalized therapeutic approach taking into consideration all these factors potentially contributing to an altered pharmacokinetic/pharmacodynamic profile could better drive safe and effective clinical use of third generation CDK4/6 inhibitors [32]. According to the application of precision medicine in the management of cancer treatment, new software, Drug-PIN, which combines data regarding DDIs and the pharmacogenomic profile of cancer patients, is under investigation at our institution [33].

#### 1.2.2. Second Line and Early Relapse

According to the Associazione Italiana di Oncologia Medica (AIOM) guidelines, “early relapse” is defined as aggressive disease that presents itself with a short disease-free interval from the adjuvant therapy (progression during or within 12 months from the end of adjuvant ET); this it is slightly different from the European Society for Medical Oncology definitions of primary and secondary resistance mentioned earlier, but it is the same definition in the three different trials:

MONALEESA-3: Patients who had a relapse during or within 12 months after completion of adjuvant or neoadjuvant ET.

MONARCH-2: Patients were required to have progressive disease while receiving neoadjuvant or adjuvant ET, within 12 months from the end of adjuvant ET.

PALOMA-3: Disease relapse or progression had to occur while on or within 12 months of completion of adjuvant therapy irrespective of menopausal status.

All these trials enrolled patients who experienced progression after mono-ET (with tamoxifen or aromatase inhibitor) and their primary endpoint was the difference in survival between CDK4/6 inhibitor plus fulvestrant versus fulvestrant plus placebo.

With the except of MONALEESA-3, both MONARCH-2, and PALOMA-3 included pre- and postmenopausal women [19,21,23,24]. According to 4th ESO-ESMO International Consensus Guidelines for Advanced Breast Cancer (ABC 4) [34], young women with ER+ ABC should have adequate ovarian suppression or ablation (OFS/OFA) and then be treated in the same way as postmenopausal women with endocrine agents with or without targeted therapies. Since the first step is to render the patient postmenopausal, all treatment recommendations should be common to both post and premenopausal patients. Therefore, patients should be informed on the options of ovarian ablation by laparoscopic bilateral oophorectomy so that it provides definitive estrogen suppression and contraception, avoids potential initial tumor flare with a luteinizing hormone-releasing hormone agonist, and may increase eligibility for clinical trials. However, no significant difference in median OS was found between premenopausal or perimenopausal patients included in PALOMA-3 (108 patients [21%], hazard ratio, 1.07; 95% confidence interval [CI], 0.61–1.86) [35] and MONARCH-2 (114 patients [17%], hazard ratio, 0.68; 95% CI, 0.37–1.25) [21] Otherwise, median PFS and ORR were significantly higher with ribociclib than with placebo in the subgroup analysis of premenopausal patients treated previously with chemotherapy for ABC included in the MONALEESA-7 trial [36].

In the indirect comparison of the populations enrolled in the three phase 3 trials with CDK4/6 inhibitors and fulvestrant (Table 2), the PFS data were similar but the results on OS were slightly different, probably not only due to the intrinsic pharmacokinetic differences between ribociclib, palbociclib, and abemaciclib [31] but also due to the different characteristics of the patient populations (Table 2). 

For example, PALOMA-3 included patients pretreated with more than one line of therapy compared with patients included in MONALEESA-3 and MONARCH-2, where only one previous line of ET was allowed [19,21,23].

In patients with previous ET, the median PFS was significantly better in the CDK4/6 arm in MONALEESA-3, MONARCH-2, and PALOMA-3. However, in postmenopausal women pretreated with ET and postmenopausal women with early relapse, the median OS reached 40.2 months in the ribociclib group compared with 32.5 months in the placebo group (hazard ratio, 0.73; 95% CI, 0.53–1.00) [19].

In the MONARCH-2 early relapse group, median OS was improved by 9.4 months, with a median OS of 46.7 months in the abemaciclib arm and 37.3 months in the placebo arm (hazard ratio, 0.757; 95% CI, 0.606–0.945; *p* = 0.01) [21]. However, earlier separation of the curves and a numerically larger effect were observed in patients with primary ET resistance (hazard ratio, 0.686; 95% CI, 0.451–1.043) compared with patients with secondary ET resistance (hazard ratio, 0.787; 95% CI, 0.606–1.021) but no statistically significant interaction was observed.

In the PALOMA-3 trial, the median OS was not statistically significant (34.9 months in the palbociclib-fulvestrant group and 28.0 months in the placebo-fulvestrant group; HR, 0.81; 95% CI, 0.64–1.03; *p* = 0.09) [24]. However, women were enrolled regardless of menopausal status and including patients treated with more than one line of previous ET. The difference in median OS was statistically significant (39.7 months in the palbociclib group and 29.7 months in the placebo group; hazard ratio, 0.72) only among those patients with documented sensitivity (secondary resistance) to previous ET.

These results highlight the following considerations: (1) ribociclib seems to perform better in an acquired resistance setting; (2) abemaciclib seems to perform better in the primary endocrine resistance setting; (3) despite the promising results with palbociclib in pretreated patients, the data on OS are still inconclusive. Overall, the adjunct of CDK4/6 inhibitors in patients with visceral disease versus patients who did not have visceral metastasis was beneficial in the three trials.

Finally, the retrospective analysis of second-line treatment in patients who progressed on CDK4/6 inhibitors as first-line therapy also deserves mention. In a recent study among patients who progressed on palbociclib (n = 104), the most frequent next-line treatment was capecitabine (n = 21), followed by eribulin (n = 16), nab-paclitaxel (n = 15), and exemestane plus everolimus (n = 12). The median PFS with hormonal therapy or combinations (n = 32) after first-, second-, and subsequent-line palbociclib was 17.0, 9.3, and 4.2 months, respectively (*p* = 0.04); whereas the median PFS with chemotherapy (n = 70) was not reached at 4.7 and 4.1 months in patients after first-, second-, or subsequent-line therapy with palbociclib (*p* = 0.56). The authors concluded that in real-world practice, hormone therapy alone or in combination with targeted agents remains an effective option after palbociclib progression [37].

In a retrospective analysis of patients treated according to the BOLERO-2 trial with everolimus plus exemestane, 17 patients had undergone previous CDK4/6 inhibitor therapy and 16 had not. In this study, there was no significant difference in PFS (median, 5.7 months versus 4.7 months, *p* = 0.890) or OS (median, 17.8 months versus 11.4 months, *p* = 0.177) between patients who received previous therapy with CDK4/6 inhibitors and those who did not, respectively. Therefore, the combination of everolimus plus exemestane remains a good option in advanced lines of treatment [38].

Moreover, a retrospective study of 58 patients with HR+/HER2− ABC who received abemaciclib after disease progression on palbociclib was conducted. In this study, 20 patients (34%) received sequential courses of therapy, and 38 patients (66%) had at least one intervening non-CDK4/6 inhibitor regimen. Fourteen patients (24%) received abemaciclib monotherapy and 44 patients (76%) received abemaciclib in combination with an anti-estrogen, including fulvestrant (52%), an aromatase inhibitor (22%), and tamoxifen (2%) [39]. In this analysis, 20 patients (34%) had early disease progression (duration < 90 days), whereas 21 patients (36%) had treatment duration exceeding 6 months, including 10 who remained on treatment at the interim analysis (range, 181–413 days). The median PFS was 5.8 months (95% CI, 3.4–8.0). Although the results are not conclusive, this is the first multicenter experience to demonstrate that a substantial proportion of patients continue to maintain clinical benefit with another CDK4/6 inhibitor after previous CDK4/6 inhibitor, highlighting the potential for their use after CDK4/6 blockade.

## 2. Future Perspectives

It is not currently known how the different combinations of ET and targeted agents compare with each other, or with single-agent chemotherapy, or which are the best candidates as CDK4/6 inhibitors. Therefore, a better understanding of the molecular pathways that could lead to resistance to CDK4/6 inhibitor is urgently required.

With this aim, several mechanisms of resistance to CDK4/6 inhibitors and potential biomarkers are under investigation [40].

**Figure 3:**
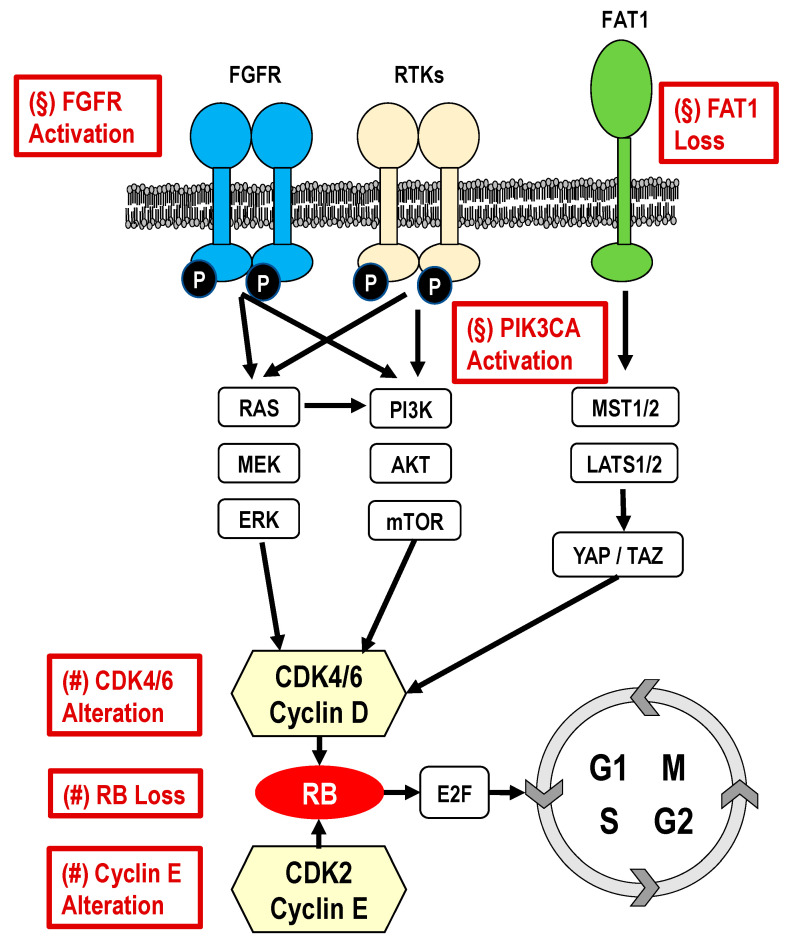
Summary of the main mechanisms potentially implicated in the resistance to CDK4/6 inhibitors: (§) Cell cycle-non-specific mechanisms; (#) Cell cycle-specific mechanisms.

Here, we present selected potential mechanisms investigated for resistance to CDK4/6 inhibitors, dividing them in two groups: (1) cell-cycle-specific mechanisms (loss of retinoblastoma [RB]; CCND1 amplification and/or loss of p16; CCNE1/2 amplification/overexpression; CDK4/6 amplification/overexpression) [41,42,43] and (2) cell-cycle-nonspecific mechanisms (PIK3CA pathway activation; FGFR pathway activation; mouse double minute 2 homolog (MDM2) overexpression; ESR1 expression and mutation; PD-1 expression; thymidine kinase-1 (TK1); FAT1 loss [41]; autophagy activation [44]). A summary of the main mechanisms potentially implicated in the resistance to CDK4/6 inhibitors is reported in Figure 3.

### 2.1. Cell-cycle-Specific Mechanisms of Resistance to CDK4/6i

- *RB loss*

RB, a tumor suppressor, represents the main target of CDK4/6. Loss of RB emerged in preclinical studies as a driver of resistance to CDK4/6 inhibitors. The incidence of loss of RB in ER+ breast cancer is low (<5%); in the PALOMA-3 study, polyclonal *RB1* mutations were identified in 4.7% of patients (palbociclib arm). No significant interaction was found between treatment and expression levels of *RB1* (and CDK4/6, cyclin D1) [24].

In the PALOMA-2 study no interaction was found between treatment and RB detected by immunohistochemistry, fluorescence in situ hybridization, or gene expression analysis (tumor samples) [45].

Total RB expression as assessed by immunohistochemistry was positive in 90.9% and negative in 9.1% of patients. Exploratory assessments of PFS by *RB1* gene using this approach revealed a consistent benefit with palbociclib plus letrozole (RB-positive tumors: hazard ratio, 0.543; *p* < 0.0001 in favor of palbociclib) with no evidence of benefit with palbociclib in truly RB-null tumors (RB-negative tumors: hazard ratio, 0.868; *p* = 0.698), but this is clearly limited by the small number of patients in this cohort).

- *CCND1 amplification/p16 loss*

CCND1 amplification (around 15% of breast cancers) and loss of p16^INK4A^ (a tumor suppressor of cyclin D1) have been hypothesized as reasonable factors of CDK4/6 inhibitors resistance. PALOMA-1 study failed to show any significant difference in PFS in patients with a loss of p16 or CCND1 amplification compared with unselected patients [22]. Moreover, expression levels of cyclin D1 were not associated with benefit from palbociclib in PALOMA-3 [24] and PALOMA-2 studies (also for p16 loss) [45].

- *CCNE1/2 amplification/overexpression*

CCNE1, the gene encoding cyclin E1, seems to be upregulated in preclinical models with resistance to CDK4/6 inhibitors [46]. However, in MONALEESA-2 trial, the mRNA expression analysis of tumor samples obtained from 391 of 668 randomized patients showed that the addition of ribociclib to letrozole provided a PFS benefit regardless of expression of *CCNE1* [17]. Also, the analysis conducted in PALOMA-2 failed to demonstrate an association between *CCNE1/2* expression (by immunochemistry, fluorescence in situ hybridization, or gene expression analysis) and benefit from palbociclib [45].

In PALOMA-3 study, tumor tissue mRNA profiling of 302 patients (from 521 randomized patients) was performed [35]. Although all biomarker groups derived benefit from palbociclib, those with low tumor *CCNE1* expression had a greater response (median PFS with palbociclib plus fulvestrant versus fulvestrant was 14.1 months versus 4.8 months) than those with high *CCNE1* expression (7.6 months versus 4.0; respectively)]. The predictive power of *CCNE1* mRNA seems to be stronger in metastatic biopsies (interaction *p* < 0.001) than archived primary biopsy samples (interaction *p* = 0.09) [35]. Similar results were reported in the MONARCH-2 study [47].

- *CDK4/6 amplification/overxpression*

*CDK4* and *CDK6* overexpression was reported to promote resistance to CDK4/6 inhibitors in preclinical models. With regard to the clinical setting, in the PALOMA-2 trial, *CDK4* expression levels predicted resistance to placebo plus letrozole but not to palbociclib plus letrozole [45]. In the letrozole arm, high levels of *CDK4* mRNA were associated with more rapid progression. *CDK6* expression did not have a similar effect (lower expression: hazard ratio, 0.596; higher expression: hazard ratio, 0.592).

In MONALEESA-2 study, the benefit of addition of ribociclib to letrozole seems to be slightly greater in patients with high versus low expression of cell-cycle-control genes (hazard ratio, 0.45 versus 0.66) [17]. In the placebo arm, the median PFS was shorter in patients with high expression of cell-cycle-control genes compared with low expression. The cell-cycle-control genes analyzed in this trial were *CCNA2, CCND1, CCND2, CCND3, CCNE1, CDK2, CDK4, CDK6, CDKN1A, CDKN1B, CDKN2A, CDKN2B, CDKN2C, RB1, E2F1, E2F3, TFDP1, and TP53*.

### 2.2. Cell-cycle-Nonspecific Mechanisms of Resistance to CDK4/6 Inhibitors

- *PIK3CA pathway activation*

In the MONALEESA-7 trial, circulating tumor DNA (ctDNA) sequencing analysis performed on 565 patients (from 672 patients randomized) showed *PIK3CA* mutations in 28% of patients [48]. The median PFS benefit with ribociclib was numerically greater in patients with wild-type *PIK3CA* than mutated *PIK3CA*, but the interaction test was not statistically significant. Similarly, the biomarker analysis of baseline ctDNA in MONALEESA-3 and MONARCH-2 studies demonstrated consistent benefit from ribociclib and abemaciclib, respectively, irrespective of *PIK3CA* status [49,50].

In MONALEESA-2 study, mRNA expression data from baseline tumor samples of the PI3K pathway genes (*AKT1, AKT2, PIK3CA*, and *PTEN*) suggested that ribociclib prolonged PFS regardless of *PIK3CA* gene pathway expression (high versus low) [17].

In PALOMA-3 trial, baseline plasma samples of 395 patients (76% of randomized patients) were available for ctDNA analysis. *PIK3CA* mutations were detected in ctDNA of 129 patients (33%) with no significant association observed with outcome [25]. The ctDNA sequencing analysis was performed on 195 patients (comparing baseline and end-of-treatment status). At baseline, 39 *PIK3CA* variants were found in 19.0% of patients; at the end of treatment, there were 55 *PIK3CA* variants in 26.7% of patients. Therefore, 7.7% of patients acquired *PIK3CA* mutations during treatment. However, the proportion of patients acquiring newly detectable *PIK3CA* mutations did not differ between the treatment groups [51]. Thus, it cannot be concluded that PIK3CA is clearly a significant predictive biomarker of response o resistance to CDK4/6 inhibitors.

Similarly, biomarker analysis in MONALEESA-3 study demonstrated consistent benefit from ribociclib plus fulvestrant, irrespective of PIK3CA alteration status, as detected in baseline ctDNA.

- *FGFR pathway activation*

In *vitro* experiments in ER+ breast cancer cells suggested that FGFR/ FGF pathway alterations are associated with fulvestrant resistance as well as cross-resistance to Palbociclib [52]. In PALOMA-2, tumors with increased expression of FGFR2 appeared to be associated with greater gain in PFS from the combination of palbociclib plus letrozole (even though the interaction test was not statistically significant) [45].

Moreover, the FGFR signaling pathway seems to have a potential prognostic role. In MONALEESA-2, ctDNA analysis showed that patients with *FGFR1* amplification (5%) had a shorter PFS compared with patients with wild-type *FGFR1* [53].

Similarly, in PALOMA-3, *FGFR1* amplification (ctDNA) identified patients at risk of early disease progression [54].

- *MDM2 overexpression*

The MDM2 downregulates p53 activity inhibiting the cellular senescence. The interruption of the senescence pathway, induced by the MDM2 overexpression, is supposed to be a mechanism of resistance to CDK4/6 inhibitors. In preclinical models, the combination of MDM2 and CDK4/6 inhibitors showed a synergistic effect [55]. In this regard, MDM2 inhibitors may overcome resistance to CDK4/6 inhibitors [56].

- *ESR1 expression and mutation*

The role of *ESR1* expression and mutation in patients receiving ER targeted therapy seems to be mainly prognostic. In MONALEESA-2, benefit from the addition of ribociclib seems to be greater in patients with high versus low ESR1 expression. However, there was a trend for longer PFS with high *ESR1* expression in both arms [17]. Similar to the PALOMA-2 study, high *ESR1* expression levels were associated with longer PFS in both arms [45].

In PALOMA-3, the benefit from palbociclib was seen despite the *ESR1* mutation status (interaction *p* = 0.74) [51]. Similar results were found in the MONARCH-2 study [50]. Thus, we cannot conclude that *ESR1* is a predictive factor for CDK4/6 inhibitors response or resistance.

- *PD-1 expression*

In PALOMA-2 study, lower PD-1 levels were associated with greater benefit with palbociclib plus letrozole [45]. Therefore, the PD-1 signaling pathway could be associated with reduced benefit in PFS from the addition of CDK4/6 inhibitors to ET. Ongoing studies are now evaluating immune checkpoint inhibitors targeting PD-1 in combination with CDK4/6 inhibitors.

- *Thymidine kinase-1 (TK1)*

Another useful factor as a prognostic biomarker of sensitivity or early response to ET is TK1, an enzyme in the pyrimidine salvage pathway that plays a crucial role in DNA synthesis/cell proliferation. In primary breast cancer tissue, high TK1 levels and activity (TKa) correlate with a poor prognosis. A pilot study of patients treated with ET showed that those with low baseline levels of plasma TKa had a better median PFS than those with high baseline levels [57]. In addition, preclinical studies suggest TKa as an early marker of proliferative inhibition in response to palbociclib [58].

- *FAT1 loss*

*FAT1* is a tumor suppressor gene, mutated in many cancers. A genomic analysis of 348 ER+ breast cancers treated with CDK4/6 inhibitors showed that loss of FAT1 (or RB1) is associated with clinical resistance to these drugs [59]. Indeed, loss of FAT1 increases CDK6 expression via the Hippo pathway, reducing sensitivity to CDK4/6 inhibitors.

- *Autophagy activation*

Preclinical evidence suggests that autophagy activation may be implicated in resistance to CDK4/6 inhibitors, given that the cell cycle arrest induced by these inhibitors can be reversed by autophagy. Therefore, the inhibition of autophagy may increase the efficacy of CDK4/6 inhibitors and may contribute to overcome resistance to these drugs [60].

### 2.3. Other Potential Mechanism of Resistance

Finally, in the ongoing BIOITALEE study, the primary objectives were to identify ctDNA alterations, their evolution at different time points of therapy, and their possible association with clinical outcome [61].

The preliminary analysis reported several tumor molecular alterations at baseline and their correlation with clinical features and outcome. In detail, copy number gains of *FGFR1-2-3* were more frequent in patients with visceral metastases; *MYC* gain or alterations in the ER nuclear function pathway were more frequent in patients with progesterone– and Ki67 ≥ 14% breast tumors. *MYC* gain, *TP53* mutations, and alterations in the HER and CDK4/6 pathways were associated with disease progression at the first tumor evaluation (10%), suggesting that they may be potential markers of intrinsic resistance to ribociclib and letrozole.

### 2.4. Strategies after CDK4/6 Inhibitor Progression and New Therapeutic Combinations

New treatment options at progression on a CDK4/6 inhibitor are emerging (Table 3 and Table 4).

The main strategies can be summarized in four groups: (1) maintain the CDK4/6 inhibitor and switch ET; (2) maintain ET and switch to another CDK4/6 inhibitor or another targeted agent; (3) maintain ET and CDK4/6 inhibitor + target a collateral pathway; (4) new agents.

In preliminary analysis of the phase 1/2 TRINTI-1 study in men and postmenopausal women with HR+, HER2− locally advanced or metastatic breast cancer following progression on a CDK4/6 inhibitor, triplet therapy with ribociclib in combination with everolimus + exemestane demonstrated 39% clinical benefit within 24 weeks [48]. Thus, TRINITI-1 met its primary efficacy endpoint and is the first trial to demonstrate both activity and tolerability of continuous triplet therapy with ET + mTORi + CDK4/6.

Around 40% of patients with HR+, HER2– ABC present an activating tumor mutation of PIK3CA. On the basis of the phase 3 SOLAR-1 study, alpelisib + fulvestrant is a potential new treatment option for patients with PIK3CA-mutant HR+, HER2– ABC who have progressed on previous ET (with/without a CDK4/6 inhibitor). In patients with *PIK3CA*-mutant disease, median PFS was 11.0 months versus 5.7 months in wild-type patients (hazard ratio, 0.65; *p* = 0.00065) [62].

However, alpelisib was recently approved in combination with fulvestrant for the treatment of postmenopausal women and men with HR+, HER2− ABC with a PIK3CA mutation after disease progression following ET as monotherapy. Therefore, the choice for patients with *PIK3CA*-mutant disease who progress after first-line therapy with CDK4/6 inhibitor plus ET is still unknown.

ByLieve, a phase 2 multicenter, open-label, two-cohort, noncomparative study of alpelisib + fulvestrant or letrozole, including patients who progressed on or after CDKi + AI or fulvestrant (metastatic setting) or with ≤1 line of previous chemotherapy (adjuvant or advanced setting) with the primary endpoint of patients alive and without disease progression at 6 months, will be determinant to this question. Although at the first interim analysis, the fulvestrant cohort seems to be better than the letrozolo cohort, the data are still immature [63].

Finally, given the likelihood of the emergence of driver mutations in the PIK3CA gene secondary to previous ET + CDK4/6 inhibitors, combined PI3K or FGFR inhibitors with CDK4/6 inhibitors, or with other target agents in triplet combinations, seems to be a promising strategy to overcome resistance to single-agent CDK4/6 inhibitors for the early phase of HR+ HER2− breast cancer (Table 5).

**Figure 4:**
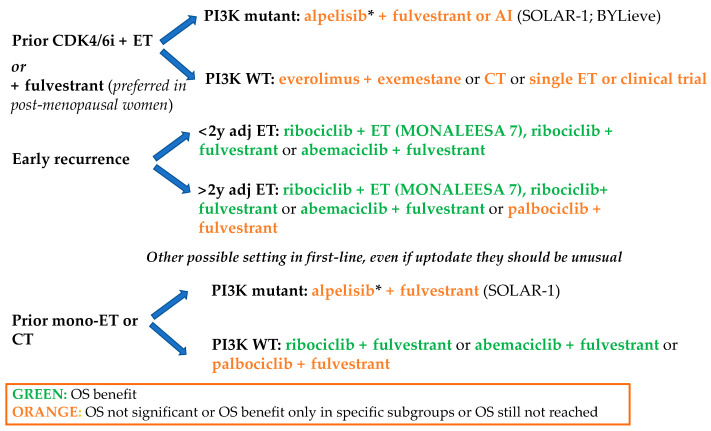
Proposed algorithm of HR+, HER2− advanced breast cancer treatment. Abbreviations: CT, chemotherapy; ET, endocrine therapy with aromatase inhibitor or tamoxifen ± ovarian suppression; AI, aromatase inhibitor. *Alpelisib is recently approved only after disease progression following endocrine therapy as monotherapy.

Despite the remarkable advances in scientific knowledge, the optimal treatment algorithm in HR+ ABC is still uncertain. However, a strategy based on (1) which agents where used previously, (2) the burden of the disease, (3) the patient’s preference, (4) costs and availability seems to be the sensible choice. Obviously, as the HR+ ABC treatment landscape evolves to include CDK4/6 inhibitors + ET as first-line therapy, later-line therapies will need to address those patients who have progressed during or after CDK4/6 inhibitor + ET (Figure 4).

Moreover, the potential mechanisms of resistance to CDK4/6i suggested by preclinical genomic and transcriptomic analyses of pathways deserve further clinical investigation (larger patient datasets and prospective studies). Also, analysis of single nucleotide polymorphisms (metabolic biomarkers), the most common type of genetic variation, may add information to predict response to CDK4/6 inhibitors at the patient level [31].

Furthermore, body composition parameters may influence the prognosis in patients receiving CDK4/6 inhibitors. In a retrospective study, baseline sarcopenia (skeletal muscle index <40) was retrospectively associated with a significantly worse PFS, whereas a high visceral fat index and higher visceral fat density were associated with better PFS [64].

Finally, the analysis of tumor tissue (still the standard) should be integrated with liquid biopsy, and longitudinal ctDNA monitoring can potentially profile the baseline mutational landscape, as well as emerging genomic clonal/subclonal mechanisms of resistance (and possibly actionable) under selective pressure of CDK4/6 inhibitors.

## 3. Conclusions

CDK4/6 inhibitors radically changed the treatment of HR+/HER2− metastatic breast cancer, resulting in a benefit in key clinical outcomes with a manageable safety profile. In this review, we reported the mechanism of action of CDK4/6 inhibitors and their implications in current clinical practice. However, intrinsic or acquired resistance can cause disease progression in a large number of patients and the understanding of the mechanism of resistance is an urgent clinical need. We described the potential mechanism of resistance and the forthcoming strategies to enhance CDK 4/6 inhibitors efficacy in improving survival and quality of life of patients. Redefining therapeutic algorithms taking into account the basal genomic profiles and the selective pressure that may occur during therapy will be the best way to personalize cancer care in patients with HR+, HER2− ABC. Although it could be interesting, whole genomic evaluation is feasible only in experimental clinical trials. But evaluation of single gene mutation, such as PIK3CA, is a close and important evaluation because targetable treatment is available and thus is soon to be introduced into clinical practice. Further research strategies on studies investigating the best sequence or the reported outcomes of triplet therapies, always balancing efficacy and safety, are also awaited.

## Figures and Tables

**Table 1:** CDK4/6 inhibitors phase 3 trials according to endocrine sensitivity/resistance patients representation and outcome results.

Drug	Trial	Setting	Endocrine Sensitivity/Resistance (%)				Efficacy		Adverse Events of Interest
			De Novo	Late Relapse	Early Relapse	Second Line	PFS (Months)	OS (Months)	
Ribociclib	MONALEESA-2 [17]	First line	34	64.7	—	—	RIBO + LET: 25.3 PBO + LET: 16.0 (HR, 0.56; 95% CI, 0.43–0.72; *p* < 0.001)	Immature	G ¾ neutropenia: 62% Diarrhea: 2.4%; TE: 0.6%; QTcF prolongation: 3.6%
	MONALEESA-7 [18]	First and second line	40	52.5	—	14 (after CT)	RIBO + TAM/NSAI: 23.8 months PBO + TAM/NSAI: 13.0 months (HR, 0.55; 95% CI, 0.44–0.69; *p* < 0.0001)	HR, 0.712; 95% CI, 0.535–0.948; *p* = 0.00973	G 3/4 neutropenia: 60.6% Diarrhea: 1% TE: NR QTc prolongation: 7%
	MONALEESA-3 [19]	First and second line	20	29	28	20	RIB + FUL: 20.5 (33.6 in first line) PBO + FUL: 12.8 (19.2 in first line) (HR, 0.593; 95% CI, 0.480–0.732; *p* < 0.001)	HR, 0.724; 95% CI, 0.568– 0.924; *p* = 0.00455	G 3/4 neutropenia: 53.4% Diarrhea: 0.6% TE: NR QTcF prolongation: 5.6%
Abemaciclib	MONARCH-3 [20]	First line	41.2	58.8	—	—	ABE + NSAI: 28.18 months PBO + NSAI: 14.76 months (HR, 0.540; 95% CI, 0.418–0.698; *p* = 0.000002)	Immature	G 3/4 neutropenia: 23.9% Diarrhea: 82.3% TE: 4.9% QTcF prolongation: 0.3%
	MONARCH-2 [21]	Second line	—	—	60	38	ABE + FUL: 16.4 PBO + FUL: 9.3 (HR, 0.553; 95% CI, 0.449–0.681; *p* < 0.001)	HR, 0.757; 95% CI, 0.606–0.945; *p* = 0.0137	G 3/4 neutropenia: 23.9% Diarrhea: 82.3% TE: 0.9% QTcF prolongation: 0.3%
Palbociclib	PALOMA-2 [22]	First line	37.6	40.01	—	—	PAL + LET: 24.8 PBO + LET: 14.5 (HR, 0.58; 95% CI, 0.46–0.72; *p* <0.001)	Immature	G 3/4 neutropenia: 66.4% Diarrhea: 1.4% TE: 0.9% QTcF prolongation: 0%
	PALOMA-3 [23,24,25]	Second line	—	—	21	79	PAL + FUL: 9.5 PBO + FUL: 4.6 (HR, 0.46; 95% CI, 0.36–0.59; *p* < 0.0001)	HR, 0.81; 95% CI, 0.64–1.03; *p* = 0.09	G 3/4 neutropenia: 62% Diarrhea: 0% TE: 1.7% QTcF prolongation: <1%

Abbreviations: AE, adverse event; PFS, progression-free survival; HR, hazard ratio; RIBO, ribociclib; LET, letrozole; PBO, placebo; FUL, fulvestrant; PAL, palbociclib; TAM, tamoxifen; NSAI, nonsteroidal aromatase inhibitor; TE, thromboembolic event.

**Table 2:** Phase 3 trials of CDK4/6 inhibitors that included patients(n) in second-line and early relapse settings, in respect to the total populations enrolled (N).

Trial	Population	n/N	Treatment	PFS (Months)	OS (Months)	ORR (%)
MONALEESA-3	Postmenopausal women and men; Second-line/early relapse subgroup	346/726	Ribociclib plus fulvestrant versus placebo + fulvestrant	14.6 (HR, 0.57)	40.2 (HR, 0.73)	40.9
MONALEESA-7	Premenopausal women; Early relapse and previous first line of CT	94/672	Ribociclib plus ET plus goserelin versus placebo + ET and goserelin	16.6 (HR 0.54)	NR (HR 0.67)	26
MONARCH-2	Pre/postmenopausal women and men; Second line/early relapse; Primary and secondary ET resistance	669	Abemaciclib plus fulvestrant versus placebo + fulvestrant	16.4 (HR, 0.55)	46.7 (HR 0.75)	48.1
PALOMA-3	Pre/postmenopausal women and men; Early relapse/second and subsequent lines; Sensitivity to ET yes/no	521	Palbociclib plus fulvestrant versus placebo + fulvestrant	11.2 (HR, 0.50)	34.9 (HR, 0.81; NS, *p* = 0.09)	25

Abbreviations: PFS, progression-free survival; OS, overall survival; ORR, overall response rate; HR, hazard ratio; ET, endocrine therapy; NS, not significant.

**Table 3:** Ongoing studies based on CDK4/6 inhibitors beyond progression.

Trial	Phase	Study Arms	Previous CDK4/6
MAINTAIN (NCT02632045)	2	Ribociclib + fulvestrant versus placebo + fulvestrant	AI + palbociclib/ribociclib
NCT02738866	2	Palbociclib + fulvestrant	Palbociclib + AI
PACE (NCT03147287)	2	Fulvestrant versus fulvestrant + palbociclib versus ± avelumab	CDK4/6 inhibitor-based regimen
NCT02871791	½	Palbociclib + everolimus + exemestane	CDK4/6 inhibitor-based regimen
TRINITI-1 (NCT02732119)	1/2	Ribociclib + everolimus + exemestane	CDK4/6 inhibitor-based regimen
NCT01857193	1B	Ribociclib + exemestane versus ± everolimus	Naive or refractory to CDK4/6-inhibitor
PALMIRA (NCT03809988)	2	Palbociclib + ET	Had clinical benefits with palbociclib + ET in first line

Abbreviations: AI, aromatase inhibitor; ET, endocrine therapy.

**Table 4:** New agents after CDK4/6 inhibitor progression.

Trial	Phase	Study Arms	Previous CDK4/6
CDK7 inhibitor after CDK4/6 inhibitor progression			
NCT03134638	1	SY-1365 + fulvestrant	CDK inhibitor + AI
NCT03363893	1	CT-7001 + fulvestrant	CDK inhibitor
CDK2 inhibitor after CDK4/6 inhibitor progression			
NCT03519178	1/2A	PF-06873600 versus ± ET	CDK inhibitor + ET
Selective estrogen receptor downregulator (SERD) (elacestrant) after CDK4/6 inhibitor progression			
EMERALD (NCT03778931)	3	Elacestrant	CDK inhibitor + AI or fulvestrant
BCL-2 inhibitor (venetoclax) afterCDK4/6 inhibitor progression			
VERONICA (NCT03519178)	2	Venetoclax + fulvestrant	CDK inhibitor-based regimen
Fibroblast growth factor receptor (FGFR) inhibitor after CDK4/6 inhibitor progression			
NCT03238196	1	Fulvestrant + palbociclib + erdafitinib	Previous palbociclib allowed
Immune checkpoint inhibitor after CDK4/6 inhibitor progression			
PACE (NCT03147287)	2	Fulvestrant versus fulvestrant + palbociclib versus ± avelumab	CDK4/6 inhibitor-based regimen
NCT0329469	1	Fulvestrant + ribociclib + PDR001	Not specified
MORPHEUS HR+BC (NCT03280563)	1/2	Fulvestrant Atezolizumab + entinostat Atezolizumab + fulvestrant Atezolizumab + ipatasertib Atezolizumab + ipatasertib+ fulvestrant Atezolizumab + bevacizumab + ET Atezolizumab + abemaciclib + fulvestrant	CDK4/6 inhibitor in first or second line

Abbreviations: AI, aromatase inhibitor; ET, endocrine therapy.

**Table 5:** Ongoing studies of multiple combination treatments with CDK4/6 inhibitors.

Trial	Treatment Arms	Phase	Study Population	Primary Endpoint	Status
Study of AZD2014 and palbociclib in patients with estrogen receptor positive (ER+) metastatic breast cancer (PASTOR)	Vistusertib (mTOR inhibitor) + palbociclib + fulvestrant Placebo + palbociclib + fulvestrant	1/2	ER+ locally advanced or MBC in postmenopausal patients pretreated with hormonal therapy	PFS	Completed no results posted
Copanlisib, letrozole, and palbociclib in treating patients with hormone receptor positive HER2 negative stage I-IV breast cancer	Copanlisib (PI3K inhibitor) + letrozole Copanlisib + letrozole + palbociclib	1b/2	ER+/HER2− postmenopausal any stage breast cancer	Change in Ki-67, DLT	Recruiting
Ipatasertib plus Palbociclib and fulvestrant versus placebo plus Palbociclib and fulvestrant in hormone receptor positive and HER2 negative locally advanced unresectable or metastatic breast cancer (IPATunity150)	Ipatasertib (AKT inhibitor) + palbociclib + fulvestrant versus placebo + palbociclib + fulvestrant	1b/3 randomized	HR+ HER2− ABC progressed during adjuvant ET or the initial 12 months of first-line ET	PFS	Recruiting
Ribociclib in combination with everolimus (RAD001) and exemestane in the treatment of postmenopausal women with hormone receptor positive, HER2 negative locally advanced or metastatic breast cancer	Ribociclib, exemestane and everolimus versus ribociclib and exemestane	Nonrandomized, two arms, parallel assignment	Recurrence while on, or within 12 months of end of, adjuvant treatment with letrozole or anastrozole, or progression while on, or within 1 month of end of, letrozole or anastrozole treatment for ABC	DLT and DCR	Completed
LEE011 in combination with fulvestrant and alpelisib or buparlisib in the treatment of postmenopausal women with hormone receptor positive, HER2 negative locally recurrent or advanced metastatic breast cancer	LEE011 + fulvestrant + alpelisib or LEE011 + fulvestrant + buparlisib or LEE011 + fulvestrant	1b/2 nonrandomized	HR+, HER2− locally recurrent or advanced metastatic breast cancer	DLT/PFS	Completed
A study to assess the tolerability and clinical activity of gedatolisib in combination with palbociclib/letrozole or palbociclib/fulvestrant in women with metastatic breast cancer	Gedatolisib + palbociclib/letrozole or gedatolisib +palbociclib/fulvestrant	1b nonrandomized	HR+, HER2− locally recurrent or advanced metastatic breast cancer	DLT ORR	Recruiting
Abemaciclib in combination with therapies for patients with metastatic breast cancer	Abemaciclib + letrozole or anastrozole or tamoxifen or exemestane or everolimus or trastuzumab or fulvestrant or pertuzumab	1b	Metastatic breast cancer	DLT	Active, not recruiting
PIPA: combination of PI3 kinase inhibitors and palbociclib with the subsequent addition of fulvestrant in PIK3CA-mutant breast cancers (PIPA)	Palbociclib + taselisib or pictilisib (+ fulvestant)	1b	Malignant solid tumors; ABC: ER+ progressed on at least one line of previous ET, or PIK3CA mutant breast cancer progressed on at least one line of previous ET or CT, breast cancer refractory to standard treatment	DLT safety	Active, not recruiting

Abbreviations: MBC, metastatic breast cancer; PFS, progression-free survival; DLT, dose-limiting toxicity; ET, endocrine therapy; DCR, disease control rate; CT, chemotherapy.
